# Non-alcoholic fatty liver disease (NAFLD) and mental illness: Mechanisms linking mood, metabolism and medicines

**DOI:** 10.3389/fnins.2022.1042442

**Published:** 2022-11-15

**Authors:** Anwesha Gangopadhyay, Radwa Ibrahim, Karli Theberge, Meghan May, Karen L. Houseknecht

**Affiliations:** Department of Biomedical Sciences, College of Osteopathic Medicine, University of New England, Biddeford, ME, United States

**Keywords:** metabolic liver disease, antipsychotic, insulin resistance, inflammation, schizophrenia, depression, obesity, ferroptosis

## Abstract

Non-alcoholic fatty liver disease (NAFLD) is the most common cause of chronic liver disease in the world and one of the leading indications for liver transplantation. It is one of the many manifestations of insulin resistance and metabolic syndrome as well as an independent risk factor for cardiovascular disease. There is growing evidence linking the incidence of NAFLD with psychiatric illnesses such as schizophrenia, bipolar disorder and depression mechanistically *via* genetic, metabolic, inflammatory and environmental factors including smoking and psychiatric medications. Indeed, patients prescribed antipsychotic medications, regardless of diagnosis, have higher incidence of NAFLD than population norms. The mechanistic pharmacology of antipsychotic-associated NAFLD is beginning to emerge. In this review, we aim to discuss the pathophysiology of NAFLD including its risk factors, insulin resistance and systemic inflammation as well as its intersection with psychiatric illnesses.

## Introduction

**FIGURE 1:**
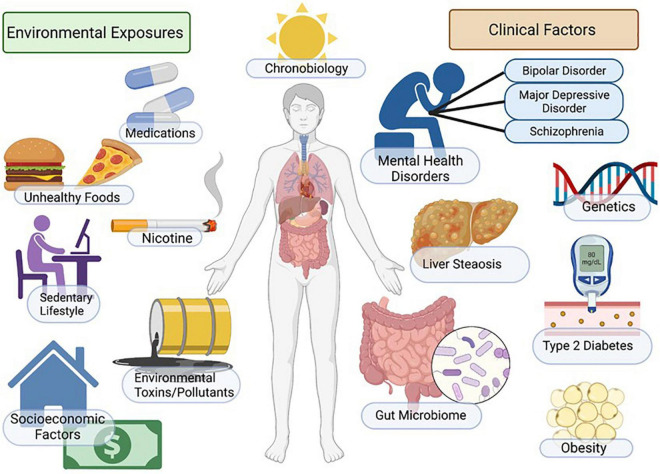
Factors regulating mood, metabolism, and NAFLD. Incidence of NAFLD in patients with mental illness is high and myriad factors contribute to increased disease risk. Patients with depression and mood disorders are more likely to be impacted by socio-economic stress, housing insecurity, poor access to healthcare, poor diet, smoking/nicotine consumption, sedentary lifestyle and sleep/circadian disruption. Additional environmental factors include genetic factors, exposure to environmental toxins and increased metabolic risk associated with psychotropic medications. Outcomes include increased adiposity, insulin resistance/diabetes, NAFLD/NASH and increased cardiovascular disease risk. Figure created with BioRender.com.

Non-alcoholic fatty liver disease (NAFLD), sometimes referred to as metabolic dysfunction associated fatty liver disease (MAFLD), represents the metabolic syndrome of the liver. NAFLD and its progressive form, non-alcoholic steatohepatitis (NASH), is a multi-system progressive disease with associated increased risk of cardiovascular disease, diabetes, osteoporosis, chronic kidney disease and cancer (Anstee et al., 2013; Adams et al., 2017; Rosato et al., 2019; Byrne and Targher, 2020; Ala, 2021; Loosen et al., 2022). Emerging evidence indicates that NAFLD is highly comorbid in patients with mental illness across the lifespan, and patients with mental illness may have unique/additional risk for NAFLD due to genetic, environmental and socioeconomic factors including exposure to psychotropic medications (Figure 1). Indeed, the increased morbidity and mortality associated with psychiatric illness is largely due to significantly increased immunometabolic disease in this patient population, including metabolic liver diseases (De Hert et al., 2018; Penninx and Lange, 2018; Mazereel et al., 2020; McIntyre et al., 2020; Lavagnino et al., 2021). Although there is strong clinical evidence that NAFLD/NASH is comorbid with psychiatric illness (Soto-Angona et al., 2020), the underlying mechanisms are not clearly defined and guidelines for patient monitoring, diagnosis and treatment paradigms for NAFLD in psychiatry have not been established. In this review we will focus on the myriad factors that may impact the development of NAFLD in patients with mood disorders, with a specific focus on the role of environmental factors, including psychiatric medications, on NAFLD risk.

## Prevalence of non-alcoholic fatty liver disease/non-alcoholic steatohepatitis

NAFLD has an approximate 25% global prevalence in the adult population and is a key factor driving increased morbidity and mortality in patients with metabolic syndrome, psychiatric disorders, as well as other conditions (Younossi et al., 2016; Soto-Angona et al., 2020; Huang et al., 2021). The highest prevalence rates, around 30%, have been reported in South America and the Middle East, with the lowest rates reported in Africa at approximately 13%. The global incidence of NASH is projected to increase by approximately 56% in the next 10 years (Huang et al., 2021). This is important to recognize as NAFLD, and more specifically NASH, is the most rapidly growing non-viral cause of hepatocellular carcinoma (Dhamija et al., 2019; Huang et al., 2021).

In the United States, the highest prevalence of NAFLD in adults has been reported in Hispanics, and the lowest prevalence in Afro-American populations (Soto-Angona et al., 2020). Few studies have evaluated the influence of sex on the prevalence of NAFLD, with limited data suggesting a higher prevalence in males (Lonardo et al., 2019; Soto-Angona et al., 2020). A 2020 systemic review and meta-analysis of 93 studies found the incidence of non-obese NAFLD in the general population to be 12.1% and the incidence of lean-NAFLD in the general population to be 5.1%. Amongst the population of individuals with non-obese-NAFLD and lean-NAFLD, 36% were found to have NASH (Ye et al., 2020).

NAFLD is increasingly prevalent in children, specifically in those with obesity and genetic predisposition (Mann et al., 2018; Nobili et al., 2019). NAFLD is the leading cause of chronic liver disease in children and increases the risk for cirrhosis, liver transplants, cardiometabolic disease and early mortality (Mann et al., 2018; Draijer et al., 2019; Nobili et al., 2019). Yu and Schwimmer (2021) estimated the global prevalence of NAFLD in children to be between 5 and 10%. A higher prevalence of NAFLD was also found amongst adolescent males and Hispanic and Asian children, while a lower prevalence was found in adolescent females and Afro-American children (Yu and Schwimmer, 2021).

### Prevalence of non-alcoholic fatty liver disease in psychiatric populations

Psychiatric patients have a shorter lifespan and higher all-cause mortality than non-psychiatric population norms; the significant reduction in life span in patients with mental illness is highly associated with increased cardiovascular disease risk and metabolic syndrome/insulin resistance (Chesney et al., 2014; Pedersen et al., 2014; Vigo et al., 2016; Plana-Ripoll et al., 2019; Lavagnino et al., 2021). Emerging evidence indicates that metabolic liver disease including NAFLD/NASH, is also more prevalent in patients with bipolar disorder and schizophrenia (Soto-Angona et al., 2020; Galiano Rus et al., 2022) and may be linked to anxiety, depression and chronic stress (Youssef et al., 2013; Tomeno et al., 2015; Choi et al., 2020; Colognesi et al., 2020; Shea et al., 2021). Furthermore, the relationship between NAFLD/NASH and mood disorders may be bi-directional in nature as metabolic liver disease has been reported to be an independent risk factor for emerging anxiety and depression (Labenz et al., 2020). Finally, NAFLD/NASH has been implicated in cognitive decline associated with dementia (Cheon and Song, 2022; Hadjihambi, 2022; Mikkelsen et al., 2022), highlighting complex and yet to be clearly defined relationships among the regulation of mood/cognition, whole-body energy metabolism, and liver disease. Factors including genetic susceptibility, socioeconomic factors, insulin resistance, systemic inflammation and psychiatric medications each contribute to increased prevalence of NAFLD in psychiatric populations.

### Pediatric mental illness: Intersection with metabolic liver disease

The intersection between psychiatric illness in children and metabolic liver disease is an area that is understudied. NAFLD in children and adolescents is the most common cause of liver disease in this age group (Hatem et al., 2022) and is highly comorbid with childhood obesity (Shaunak et al., 2021). Pediatric NAFLD is influenced by myriad additional factors including genetics, low birth weight and male gender (Le Garf et al., 2021) and often presents with a distinct histopathology compared to NAFLD in adult populations (type 1 NASH), specifically steatosis with portal inflammation and/or fibrosis without per sinusoidal fibrosis and hepatocellular ballooning type 2 NASH; (Nobili et al., 2019).

Children presenting with mood disorders, behavioral disorders or developmental disorders are often treated with psychiatric medications. Psychiatric drugs are highly prescribed in pediatric populations (both on and off label) and are associated with increased incidence of adverse metabolic effects including obesity, diabetes and dyslipidemia (Harrison et al., 2012; Libowitz and Nurmi, 2021). Psychotropic medications, including antipsychotics, antidepressants and mood stabilizers are associated with increased incidence and severity of NAFLD/NASH in pediatric patients (Gracious et al., 2015; Mouzaki et al., 2019). Atypical antipsychotic (AA) medications are FDA approved for the treatment of behavioral aspects of autism, including for children as young as 2 years of age (Iasevoli et al., 2020; Persico et al., 2021), thus this patient population bears a large medication-associated metabolic burden (Libowitz and Nurmi, 2021). Children with autism spectrum disorder and Down syndrome suffer from an almost twofold incidence of obesity and threefold risk of being diagnosed with NAFLD, due to genetic and environmental factors including the use of medications such as AA, mood stabilizers and selective serotonin reuptake inhibitors SSRIs; (Shedlock et al., 2016). Additionally, high rates of off label prescribing of AA in children and youth occur, particularly to control impulsivity and aggression associated with attention deficit/hyperactivity disorder ADHD; (Correll and Blader, 2015; Sohn et al., 2016; Candon et al., 2021) and treatment-resistant depression (Meng et al., 2022). As discussed earlier, AA are associated with significant endocrine and metabolic side effects including hyperlipidemia, insulin resistance and weight gain, each of which are associated with the development of NAFLD. Children appear to be especially sensitive to these metabolic side effects (Dori and Green, 2011; Kryzhanovskaya et al., 2012; Libowitz and Nurmi, 2021; Minjon et al., 2022).

## Pathophysiology of non-alcoholic fatty liver disease: Disease progression and diagnosis

### Pathophysiology and progression

**FIGURE 2:**
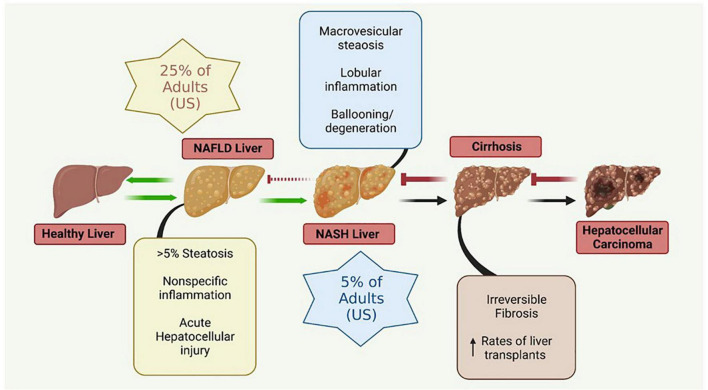
Metabolic liver disease is a multifactorial, progressive disease that if left untreated can lead to irreversible conditions necessitating liver transplantation. NAFLD, non-alcoholic fatty liver disease; NASH, non-alcoholic steatohepatitis. Figure created with BioRender.com.

The liver plays a crucial role in the regulation of many physiological systems including carbohydrate and lipid metabolism, drug metabolism, iron metabolism and immune function. NAFLD is a progressive disease that is characterized by hepatic insulin resistance and inflammation associated with fat accumulation in the liver (Figure 2). NAFLD, characterized by simple steatosis, can progress to NASH, fibrosis, cirrhosis, and ultimately hepatocellular carcinoma (Buzzetti et al., 2016). Type 2 diabetes (T2D) is often comorbid with NAFLD, and increases the risk for rapid progression to NASH and cirrhosis (Targher et al., 2021). NAFLD is associated with a significant increase in the risk of developing T2D, irrespective of obesity and other metabolic risk factors, illustrating the complexity and bidirectionality of hepatic metabolic dysregulation (Targher et al., 2021).

The simple steatosis characteristic of NAFLD is often reversible with lifestyle modifications; however, more advanced forms of NASH are not reversible. Advanced stages of NASH are thought to be triggered by multiple, synergistic mechanisms including insulin resistance, genetic and epigenetic factors, mitochondrial mutations and dysfunction, adipokines, the microbiome (gut-liver axis), and diet (Buzzetti et al., 2016).

### Diagnosis of non-alcoholic fatty liver disease/non-alcoholic steatohepatitis

Diagnosis of NAFLD/NASH often occurs in the later stages of disease, when non-surgical interventions that would ameliorate earlier disease are largely ineffective. This is due to a combination of factors including a lack of symptoms early in disease leading to an absence of clinical attention, the expense and invasiveness of effective diagnostic procedures, and the absence of circulating biomarkers (Baranova and Younossi, 2008; Piazzolla and Mangia, 2020). Liver biopsy is the current diagnostic “gold standard” for NAFLD/NASH; however, it is expensive, invasive, and not widely available in outpatient settings.

Whole liver imaging studies, both ultrasonography and magnetic resonance imaging (MRI) have been historically used as a proxy for liver biopsy. Ultrasonography, specifically transient elastography (TE), has emerged as a valuable tool for the diagnosis of NAFLD in the absence of liver biopsy. TE allows for the calculation of the controlled attenuation parameter (CAP), a determination of the “stiffness” of liver tissue, widely interpreted as a measure of hepatic fibrosis, and hepatosteatosis. The positive predictive value of CAP as determined by ultrasonography for NAFLD/NASH is 86% (de Lédinghen et al., 2016), indicating that it is a reasonably sensitive and non-invasive diagnostic measure for NAFLD/NASH. MRI-based determination of proton density fat fraction (PDFF) adds an additional capacity for staging the grades of steatosis present, suggesting that it may be of utility in monitoring NAFLD patients for progression to NASH (Kang et al., 2011; Tang et al., 2013; Middleton et al., 2017; Loomba et al., 2020).

Circulating biomarkers represent an ideal, low-cost diagnostic screening tool for NAFLD/NASH; however, highly specific and sensitive blood tests remain elusive. Liver enzymes are within normal limits in up to 80% of patients with histologically confirmed NAFLD/NASH (Browning et al., 2004), and NAFLD histopathology is not significantly different between patients whose liver enzymes are within or outside normal limits (Fracanzani et al., 2008). Taken together, these findings indicate that measurement of circulating liver enzymes is not a reliable method for detecting NAFLD or NASH until the very late stages of disease. Metabolomic studies aimed at differentiating NAFLD/NASH patients from healthy controls indicate that pyroglutamate and changes in circulating bile salts have upward of 80% diagnostic sensitivity, and may be adopted in the future (Browning et al., 2004; Fracanzani et al., 2008).

Multiple indices utilizing widely available measures to calculate a composite score for the probability of NAFLD/NASH have been developed. The NAFLD liver fat score (NLFS) incorporates metabolic syndrome, type 2 diabetes status, fasting insulin level, and fasting aspartate aminotransferase/alanine aminotransferase ratio, and has an 86% sensitivity in detecting NAFLD (Kotronen et al., 2009). The hepatic steatosis index (HSI) utilizes AST/ALT ratio, body mass index (BMI), diabetes status, and gender to determine a predictive value for NAFLD/NASH. The HSI has a 66% sensitivity for NAFLD/NASH diagnosis, but the sensitivity is lower in diabetic patients (Lee et al., 2010). The fatty liver index (FLI) utilizes BMI, waist circumference, serum triglyceride levels, and gamma-glutamyl transferase levels to predict NAFLD (Bedogni et al., 2006). Combining the NLFS, HSI, and FLI results in a 70–80% sensitivity in detecting NAFLD/NASH (Kahl et al., 2014). Given that the measures used to generate these composite scores are affordable and widely available, a combination of the NLFS, HSI, and FLI indices represent a promising strategy for NAFLD/NASH screening until novel biomarkers are readily available.

## Pharmacology of psychiatric medications: Association with metabolic liver disease

Psychotropic medications, which modify mood and behavior, include antidepressants, antipsychotics and mood stabilizers. These drug classes have been long known to alter body weight and energy metabolism (Dayabandara et al., 2017), and SSRI antidepressants and AA medications are associated with increased incidence of metabolic diseases including diabetes, NAFLD/NASH and osteoporosis (Houseknecht et al., 2017; Soto-Angona et al., 2020). AA drugs can cause rapid and significant weight gain and insulin resistance in children and adults (Dayabandara et al., 2017; Burghardt et al., 2018; Libowitz and Nurmi, 2021) thus drug-associated weight gain is considered a significant metabolic risk for patients consuming these medications. Additionally, drug associated metabolic effects may be due to factors other than weight gain as studies in preclinical models using clinically relevant doses of AA medications indicate that insulin resistance, immune dysfunction and NAFLD can occur *prior to drug associated weight gain* (Houseknecht et al., 2007; Martins et al., 2010; May et al., 2019, 2020; Beauchemin et al., 2020; Rostama et al., 2020) indicating that AA effects are multifactorial, dose-dependent and progressive. Additionally, AA effects on the liver are likely *via* central and peripheral mechanisms. For example, AA medications acutely alter hepatic glucose production and whole body insulin sensitivity *via* hypothalamic signaling mechanisms (Houseknecht et al., 2007; Martins et al., 2010; Fernø et al., 2011; Kowalchuk et al., 2019, 2021) and can directly alter hepatic lipid metabolism (Raeder et al., 2006; Oh et al., 2011).

### Mechanistic pharmacology of drug-associated non-alcoholic fatty liver disease

**FIGURE 3:**
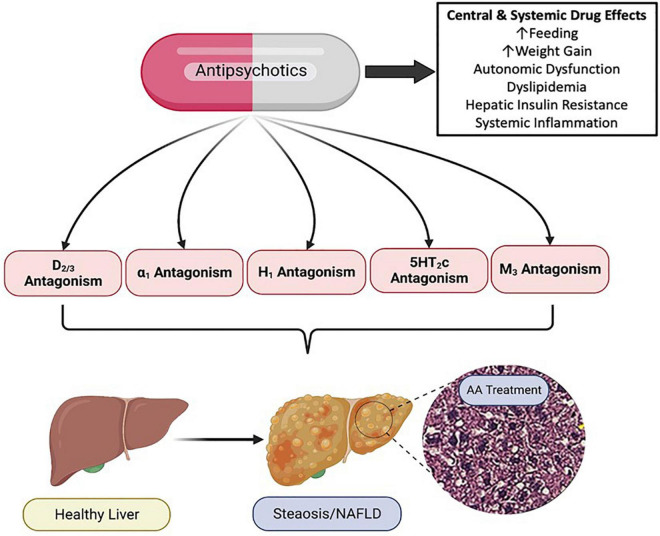
Pharmacology of Antipsychotic Induced Non-alcoholic Fatty Liver Disease (NAFLD/NASH). Antipsychotic medications have complex and diverse receptor pharmacology across the drug class, displaying potent antagonism and inverse agonism of multiple G protein coupled receptors. Antipsychotic (AA) associated metabolic side effects, including those associated with NAFLD/NASH can be mediated centrally and peripherally, and likely involve multiple organ systems and cell types. NAFLD, non-alcoholic fatty liver disease; NASH, non-alcoholic steatohepatitis; D, dopamine receptor; α, alpha adrenergic receptor; H, histamine receptor; 5HT, serotonin receptor; M, muscarinic receptor. Figure created with BioRender.com.

The mechanistic pharmacology relating to psychotropic drug induced metabolic liver disease is not well understood. Psychotropic medications have potent and diverse receptor binding characteristics. AA medications are potent antagonists of dopaminergic, serotonergic, histaminergic, alpha-adrenergic and muscarinic receptors (Siafis et al., 2018). Members of the AA drug class exhibit varying affinity for these receptor subclasses, and in some cases, receptor targeting correlates with AA side effect profiles (Figure 3; Sykes et al., 2017; Siafis et al., 2018). Furthermore, as these receptors are expressed both centrally and peripherally, it is clear that at least some of the adverse drug effects are due to antagonism of these receptors in peripheral tissues (Kimura et al., 2021). For example, adverse effects of the AA medication, risperidone, on bone biology appear to be due, at least in part, to direct effects of risperidone which distributes to the bone marrow compartment and binds to dopamine receptors expressed in bone (Motyl et al., 2017).

### Antipsychotic drugs alter autonomic nervous system function

Autonomic nervous system disruption is a unifying factor in metabolic and psychiatric disorders and antipsychotic medications have been shown to alter autonomic nervous system activity in patients with schizophrenia (Iwamoto et al., 2012; Stogios et al., 2021). As AA medications antagonize α1 adrenergic, muscarinic and dopaminergic receptors, drug associated effects on autonomic nervous system function are perhaps not surprising. The liver is regulated by both sympathetic and parasympathetic innervation, and the autonomic nervous system coordinates and regulates diverse hepatic physiology including glucose and liver metabolism, fluid balance, hepatic blood flow, bile flow, hepatic fibrosis and regeneration as well as circadian regulation of liver function (Jensen et al., 2013; Amir et al., 2020; Ishay et al., 2021; Mizuno et al., 2021). The sympathetic nervous system has also been implicated in the pathophysiology of obesity, insulin resistance/metabolic syndrome (Moreira et al., 2015; Guarino et al., 2017; Carnagarin et al., 2019; Russo et al., 2021; Yu and Lee, 2021), and emerging data from both pre-clinical and clinical studies suggest that sympathetic nervous system activation contributes to the development of NAFLD (Carnagarin et al., 2021).

Antipsychotic medications, which to varying degrees alter autonomic function, also are associated with diverse and profound metabolic and endocrine side effects. Sympathetic regulation of energy metabolism, bone biology, as well as pathways regulating cardiac and liver function (including NAFLD/NASH) are altered with AA treatment, preclinically (Savoy et al., 2010; Motyl et al., 2012, 2015; Beauchemin et al., 2020; Rostama et al., 2020; Carnagarin et al., 2021; Kunst et al., 2021). Thus, psychiatric medications which alter autonomic function likely contribute to medication-associated NAFLD in patients who consume these medications, and modulating sympathetic nervous system tone may prove to be an important therapeutic strategy for the treatment of NAFLD.

### Dopaminergic signaling

Dopamine (DA) is a neurotransmitter that plays important roles in learning, cognition and motivation/reward systems (Berke, 2018; Klein et al., 2019). Alterations in dopaminergic tone and signaling have long been implicated in metabolic syndrome/insulin resistance, are thought to be underlying mechanism(s) in the circadian regulation of metabolism (Rubí and Maechler, 2010; Liu and Borgland, 2019; Kullmann et al., 2020; Stoelzel et al., 2020), and dopamine agonists are FDA approved for the treatment of Type 2 Diabetes (Defronzo, 2011; Kabir et al., 2022). Furthermore, metabolic diseases including insulin resistance, diabetes and NAFLD are linked to cognitive dysfunction including depression and dementia (Tomeno et al., 2015; Estrada et al., 2019; Colognesi et al., 2020; Hassan et al., 2020; De Iuliis et al., 2022; Hadjihambi, 2022). Pre-clinical studies have reported that NAFLD is associated with lower concentrations of DA (and/or higher concentrations of DA metabolites) in the brain, suggesting a link between DA and NAFLD (Erbaş et al., 2015; Xu et al., 2017; Higarza et al., 2019; Mikkelsen et al., 2022).

Antipsychotic medications (both typical and atypical) are potent antagonists/inverse agonists of dopamine receptors (especially D2 receptor subtypes) as most were designed to moderate the overproduction/overactivity of dopamine in patients with psychosis (the dopamine hypothesis of psychosis) (McCutcheon et al., 2019). Published mechanistic studies exploring the role of dopamine signaling in NAFLD are limited, however, preclinical studies have illustrated that low, clinically relevant doses of AA medications can rapidly induce NAFLD/NASH in the absence of weight gain (Rostama et al., 2020) and these effects are associated with dramatic shifts in expression of the hepatic and cardiac proteomes (Beauchemin et al., 2020; Rostama et al., 2020), consistent with altered signaling pathways, including those downstream of dopamine receptors.

### Serotonergic signaling

Despite diverse pharmacology across the medication classes, SSRI and AA drugs share pharmacological targeting of serotonin signaling. SSRI antidepressants increase concentrations of the neurotransmitter serotonin by blocking the reuptake of serotonin into neurons. Conversely AA are potent antagonists of serotonin 5HT receptors (Ananth et al., 2001). Serotonin is produced centrally and also in the periphery, and is known to play a role in the regulation of hepatic energy metabolism (Stasi et al., 2019; Park et al., 2021). Serotonin signals *via* a large family of 5HT receptors (Park et al., 2021). Chronic liver diseases including NAFLD/NASH include a robust wound healing response that includes hepatic fibrosis; serotonin signaling has been implicated in the regulation of pro-fibrotinogenic aspects of hepatic liver disease (Stasi et al., 2019). Indeed, serotonin signaling *via* diverse 5HT receptors regulates various aspects of the tissue repair process and efforts are underway to determine the roles of specific 5HT receptor subtypes on these processes in order to inform therapeutic drug discovery efforts. Emerging literature suggests that SSRI medications such as fluoxetine stimulate NAFLD *via* increased serotonin production (Ayyash and Holloway, 2021) and modulation of autophagy (Niture et al., 2018).

### Histaminergic and muscarinic signaling

Antipsychotic drugs exhibit diverse receptor binding affinity for histaminergic and muscarinic receptors, with clozapine and olanzapine showing the highest affinities across the AA drug class (Bymaster et al., 1996; Richelson and Souder, 2000). Clozapine and olanzapine also have the greatest potential clinical metabolic liability in that they potently induce weight gain/obesity and increased incidence of type 2 diabetes across the lifespan when compared to other antipsychotic medications (Holt, 2019). Further evidence to support mechanistic role of H1 and muscarinic M3 receptors in AA associated metabolic effects include association of genetic variants of these receptors (two single-nucleotide polymorphisms in HRH1 and CHRM3 receptor genes) with BMI and glycated hemoglobin (HbA1c) concentrations in patients treated with antipsychotic drugs (Vehof et al., 2011).

Histamine (*via* the H1 receptor) is part of the leptin signaling pathway in the hypothalamus (Masaki et al., 2004) and antagonism of the H1 receptor is thought to result in AA associated weight gain, in the case of AA drugs with high affinity for the H1 receptor (Kroeze et al., 2003). Evidence to support direct effects of H1 receptor antagonism on development of NAFLD is scarce however, H1R antagonism was reported to exacerbate high fat diet induced NAFLD in wild-type mice (Raveendran et al., 2014), suggesting a role of this receptor subtype in the pathogenesis of NAFLD. A retrospective study conducted in a Hispanic pediatric population with NAFLD found that BMI and z-scores in children who took antihistamine medications compared to children who were not medicated (Saad et al., 2020).

Data examining the role of muscarinic receptors in the regulation of liver injury and metabolic liver disease are also limited. Acetylcholine plays a role in the regulation of fibrogenesis (Morgan et al., 2016) and muscarinic receptors (M2/3) have been reported to play a role in the development of hepatic fibrogenesis associated with NASH (Jadeja et al., 2019). Recently data are emerging that suggest that M3 receptor signaling may be protective against fatty liver disease (Jadeja et al., 2019), possibly implicating M3 receptor antagonism as a contributing factor in AA associated NAFLD/NASH. Clearly more research is needed to determine the role(s) of histamine and muscarinic receptor antagonism in medication associated metabolic syndrome and NAFLD.

### Mechanistic toxicology of atypical antipsychotic medications: Omics approaches

Given the multifaceted pathophysiology of progressive metabolic liver disease coupled with the complex pharmacology of psychotropic medications, there is a need for powerful experimental tools to identify underlying mechanisms which should inform clinical practice in psychiatry as well as inform biomarker and therapeutic development for NAFLD/NASH. Omics technologies, including genomics, epigenomics, transcriptomics, proteomics, metabolomics, lipidomics and glycomics, are high throughput analytical tools that enable bioanalysis of complex pathophysiological and pharmacological dynamics (Perakakis et al., 2020). Recent studies utilizing proteomic approaches have identified myriad signaling pathways that are disrupted by clinically relevant AA exposures in pre-clinical and clinical samples that are associated with significant changes in multiple NAFLD associated pathways including those regulating lipid metabolism, mitochondrial function, inflammation, iron metabolism and insulin signaling (May et al., 2019, 2020, 2022; Beauchemin et al., 2020; Rostama et al., 2020).

## Mechanistic synergies between non-alcoholic fatty liver disease and mental illness

### Obesity and insulin resistance

Obesity continues to be a serious and worsening global public health problem, leading to increased morbidity and mortality across the lifespan. Obesity is a multi-system disease and an important predisposing condition leading to the development of metabolic liver disease. Indeed, the severity and duration of obesity (age of onset) impact the severity and prevalence of NAFLD (Lonardo et al., 2020). Obesity is not only associated with NAFLD, but also with progressive forms of the disease including NASH, cirrhosis and hepatocellular carcinoma, indicating that obesity increases all-cause mortality as well as liver-specific mortality in patients with NAFLD (Polyzos et al., 2019).

The prevalence of obesity among adults with psychiatric disorders is approximately twice that of those without psychiatric disorders (Daumit et al., 2003; Allison et al., 2009) and adolescents with psychiatric disorders are at high risk for developing obesity (Chao et al., 2019). In patient populations seeking metabolic surgery for the treatment of obesity, psychiatric disorders including depression, anxiety and binge eating disorders were prevalent in 60–70% of patients (Malik et al., 2014; Dawes et al., 2016; Sarma et al., 2021). In psychiatric populations, the prevalence of obesity ranges from 20 to 70% for depression, bipolar disorder and schizophrenia (Holt and Peveler, 2009; Afzal et al., 2021) supporting the hypothesis of a bi-directional relationship between mood disorders and obesity (Rajan and Menon, 2017). The relationship between mental health and obesity is complex given possible predisposing genetic factors depression; (Brainstorm Consortium et al., 2018) as well as the fact that mental illness can impact nutritional choices, physical activity, and sleep, all of which in turn affect body weight regulation (Ventura et al., 2014; Vancampfort et al., 2016, 2017; Jakobsen et al., 2018; Bacaro et al., 2020). Comorbid mental illness and obesity also strongly correlate with social determinants of health, discussed further in Section “Social determinants of health.”

Insulin resistance, most commonly associated with obesity, is strongly implicated in the development of NAFLD/NASH (Fracanzani et al., 2008; Samuel and Shulman, 2018; Polyzos et al., 2019; Smith et al., 2020). Indeed NAFLD/NASH is one of many manifestations of insulin resistance and is an independent risk factor for cardiovascular disease. Insulin resistance is a multi-system disorder and leads to increased hepatic *de novo* lipogenesis as well as a reduction in insulin-inhibition of lipolysis in adipose tissue, with the net effect of increasing hepatic lipid content (Guilherme et al., 2008; Buzzetti et al., 2016; Smith et al., 2020). Adipocyte dysfunction, including dysregulation of adipokine synthesis (e.g., adiponectin, leptin) and secretion as well as alterations in inflammatory cytokine production all contribute to chronic inflammatory lipotoxicity in multiple tissues including the liver (Guilherme et al., 2008; Cusi, 2009; Marra and Svegliati-Baroni, 2018; Chang et al., 2020; Rajesh and Sarkar, 2021). Inflammatory lipotoxicity is associated with mitochondrial dysfunction, oxidative stress and endoplasmic reticulum dysfunction.

### Inflammation, oxidative stress and mitochondrial dysfunction in non-alcoholic fatty liver disease and mental illness

Systemic inflammation, characteristic of chronic diseases such as obesity and diabetes, contributes to both psychiatric disorders and NAFLD. The relationship between inflammation and metabolic liver diseases (NAFLD/NASH) is both causal and reciprocal, an example of immunometabolic disease (Katsarou et al., 2020; Chavakis, 2022). Inflammatory signals resulting from lipid peroxidation are critical to the development of NAFLD, and multiple factors contribute to the onset of hepatic lipid peroxidation. Insulin resistance is considered a primary driver of NAFLD pathophysiology, and insulin resistance induced hepatic steatosis, fatty acid overload and lipotoxicity result in hepatic vulnerability related to inflammation and oxidative stress.

**FIGURE 4:**
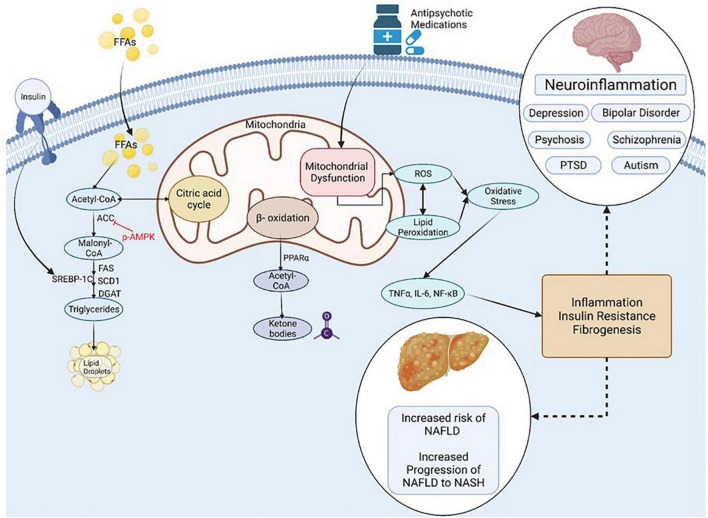
Inflammation, Oxidative Stress and Mitochondrial Dysfunction are Unifying Mechanisms underlying NAFLD and Mental Illness. NAFLD, non-alcoholic fatty liver disease; NASH, non-alcoholic steatohepatitis; PTSD, post-traumatic stress syndrome; FFA, non-esterified (free) fatty acids; ROS, reactive oxygen species; ACC, Acetyl-CoA carboxylase; DGAT, diacylglycerol acyltransferases; SCD1, Stearoyl-CoA desaturase-1; FAS, Fatty acid synthase; SREBP-1C, Sterol Regulatory Element Binding Protein 1c; PPAR, Peroxisome proliferator-activated receptor; TNF, tumor necrosis factor; IL-6, Interleukin-6; NF-kB, Nuclear Factor-Kappa B. Figure created with BioRender.com.

Mitochondria act to produce energy/ATP as well as generate reactive oxygen species (ROS) and play a prominent role in regulation of apoptosis (Figure 4). Mitochondrial remodeling and dysfunction is considered a signature of the transition from NAFLD to NASH (Sunny et al., 2017) and NAFLD/NASH has been referred to as a mitochondrial disease (see review, 161). Clinical and preclinical evidence indicate that hepatic inflammation and insulin resistance are associated with altered hepatic mitochondrial energetics including altered ATP homeostasis, increased fatty acid oxidation, increased production of ROS and lipid peroxidation in NAFLD/NASH (Caldwell et al., 1999; Cortez-Pinto et al., 1999; Sanyal et al., 2001; Sunny et al., 2017; Lee et al., 2019; Xu et al., 2019; Dabravolski et al., 2021). Furthermore, accumulating evidence points to mitochondrial mutations playing an important role in NAFLD/NASH development and progression (Dabravolski et al., 2021).

Many psychiatric disorders, including depression, schizo phrenia, autism spectrum disorder and neurodegenerative disorders are associated with chronic neuroinflammation, oxidative stress and mitochondrial dysfunction (Streck et al., 2014; Rosenblat and McIntyre, 2017; Allen et al., 2018; Filiou and Sandi, 2019; Furman et al., 2019; Beurel et al., 2020; Bjørklund et al., 2020; Resende et al., 2020; Leng and Edison, 2021; Troubat et al., 2021). Mitochondrial dysfunction in mental illness is apparent by reduced levels of ATP and increased oxidative stress in the brain of patients with clinical depression and animals in preclinical models (Gamaro et al., 2003; Moretti et al., 2003; Martins-de-Souza et al., 2012; Allen et al., 2018). Mitochondrial dysfunction is also associated with the incidence, disease progression, and clinical outcomes in schizophrenia and bipolar disorder (Maas et al., 2017; Ermakov et al., 2021).

Conversely, AA medications downregulate inflammatory responses both clinically and preclinically, (Chen et al., 2012; Al-Amin et al., 2013; Capuzzi et al., 2017; Romeo et al., 2018; May et al., 2019, 2020), and antidepressant medications including fluoxetine, desipramine and imipramine alter mitochondrial function. Administration of AA and antidepressant medications to a patient population with hyperinflammatory responses, chronic oxidative stress, and mitochondrial dysfunction as aspects of their disease holds potential to reverse these states. In this way, the pharmacological effects of these medications on inflammation, oxidative stress, and mitochondrial function may represent an underappreciated aspect of their efficacy (Adzic et al., 2013; Villa et al., 2016, 2017; Emmerzaal et al., 2021).

### Ferroptosis and non-alcoholic fatty liver disease

Associations between dysregulated iron metabolism and certain psychiatric conditions have been described. This includes both serum iron deficiency (Kuloglu et al., 2003; Kim S. W. et al., 2018) and iron overload (Cutler, 1994; Feifel and Young, 1997; Owiredu et al., 2019; Keleş Altun et al., 2021). Serum measures in psychiatric patients can be complicated by multiple treatment confounders; however, case-controlled studies with drug-naïve patients still support a direct association between psychosis and serum iron concentrations (Cao et al., 2019; Owiredu et al., 2019). Additionally, antipsychotic medications themselves can alter iron metabolism (May et al., 2022). Patients with metabolic liver disease have been reported to have altered levels of serum ferritin, hemoglobin, and hematocrit (Li et al., 2012, 2014; Bernhardt et al., 2018; Jung et al., 2019), and hemoglobin has been reported as an independent predictor of fibrosis in lean patients with NAFLD (Akyuz et al., 2015) and NASH (Nelson et al., 2007).

**FIGURE 5:**
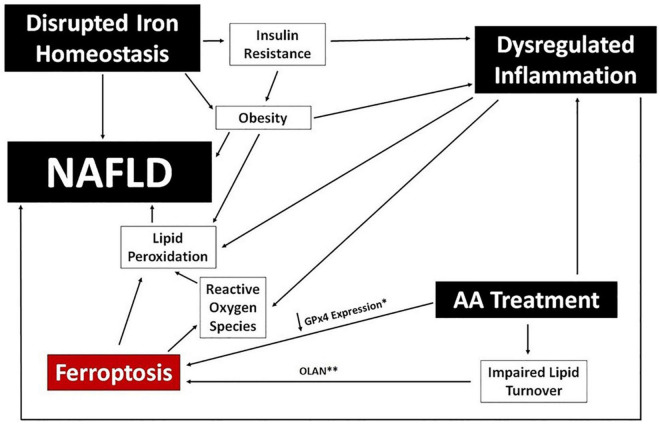
Ferroptosis and NAFLD in Psychiatric Patients. A potential mechanistic link between ferroptosis and NAFLD is proposed using clinical benchmarks reported in psychiatric patients (black boxes). Specific data points reported for certain AA medications are noted; specifically, downregulation of GPx4 expression (*) in human cells following exposure to risperidone, volanaserine, or amisulpride (Malekizadeh et al., 2020) and pathway analysis indicating impaired lipid turnover in the liver of mice treated with olanzapine (^**^) (Rostama et al., 2020).

Iron imbalance contributes directly to the development of NAFLD by stimulating lipid peroxidation and leading to the generation of ROS, and has a potential secondary link *via* association with both obesity and insulin resistance (Chitturi and George, 2003; Baptista-González et al., 2005; Manco et al., 2011; González-Domínguez et al., 2020). There is also a growing appreciation for an association between NAFLD/NASH and ferroptosis, the process of iron-dependent regulated cell death (Tsurusaki et al., 2019; Wu et al., 2021). Ferroptosis is induced by iron accumulation, a decrease in lipid turnover, and inhibition of glutathione peroxidase (GPx4), and the process itself results in further lipid peroxidation and the production of lipid-derived ROS. The extent to which ferroptosis is directly responsible for NAFLD caused by iron imbalance, or whether it simply exacerbates existing disease, is unclear. A potential associative role of ferroptosis in the development of NAFLD in psychiatric patients is depicted in Figure 5.

### Chronobiology regulates mood and metabolic liver disease

Biological systems are entrained by circadian rhythms that are mediated both centrally and peripherally. The circadian clock (CC) is an endogenous timing system that consists of a central circadian clock (CCC) located in the suprachiasmatic nucleus (SCN) as well as peripheral circadian clocks (PCC) located in various tissues including the liver (Stenvers et al., 2019). The central clock functions to communicate with organ systems in order to orchestrate and coordinate physiological processes including feeding and metabolic homeostasis (Stenvers et al., 2019). Specifically, the CC functions by receiving signals from the retina that measures ambient light, and translating those inputs to regulate transcription of myriad genes in rhythm with light/dark cycles. The cell autonomous CC operates in a feedback loop system to regulate the transcription/translation of myriad genes in response to light/dark cycles (Zhang et al., 2014; Takahashi, 2017; Kim and Lazar, 2020). Additionally, changes in feeding times stimulate metabolic sensors to modify PCC rhythms (Koch et al., 2020). When feeding takes place during the resting phase, there is a mismatch between the CC and the PCC as only the PCC is affected. As a result, there is dysregulation in metabolic processes in the liver and pancreas (Bugge et al., 2012; Stenvers et al., 2019). Indeed, hepatic physiology is regulated by circadian rhythms, at least in part *via* dopaminergic (D2) receptor signaling (Cervantes et al., 2022). Furthermore, circadian dysregulation is implicated in development of NAFLD/NASH (Mukherji et al., 2020; Saran et al., 2020). Circadian disruption of hepatic lipid metabolism leads to increased levels of free fatty acids, cholesterol and triglycerides while a reduction in the GLUT2 transporter leads to increased levels of glucose in the blood and higher levels of gluconeogenesis (Lamia et al., 2008; Bugge et al., 2012; Stenvers et al., 2019; Mukherji et al., 2020).

In addition to metabolic processes, mood and mental health are also under circadian influence. Circadian disruption is associated with mood disorders including depression and bipolar disorder (Bedrosian and Nelson, 2017; Vadnie and McClung, 2017; Ahmad et al., 2020). Alterations in the light-dark cycle which influence the CC rest-active cycle leading to cortisol-dependent mood disorders, specifically, seasonal affective disorder (Dijk et al., 2012; Ahmad et al., 2020). With changes to CC, areas of the brain responsible for mood, such as the amygdala and habenula, are activated or inhibited, inappropriately leading to changes in affect, behavior and sleep schedules (Hattar et al., 2006; Ahmad et al., 2020). Additionally, circadian regulation of hormones including cortisol (Schwartz and Klerman, 2019) is driven by light exposure meaning that global travel can have an impact on mood and sleep (Ahmad et al., 2020).

Drug efficacy and toxicology are also under circadian control; it has been long known that timing matters in terms of drug dosing, with clinically relevant, time of day effects being observed across myriad drug classes, including psychotropic medications (Nagayama, 1993; Kawai et al., 2019; Ruben et al., 2019; Nahmias and Androulakis, 2021; Walton et al., 2021). Mechanistically, these effects are due to circadian regulation of gene expression for enzymes and transporters involved in drug absorption, distribution, metabolism, and excretion, with ultimate effects on circadian pharmacokinetics and medication chrono efficacy and chrono toxicity (Bicker et al., 2020; Lu et al., 2020; Nahmias and Androulakis, 2021). Given the high rate of consumption of psychiatric medications world-wide, and the medication associated effects on energy metabolism and prevalence of metabolic diseases including NAFLD, the further impact of circadian regulation of psychiatric drug effects deserves further investigation.

### Genetic and epigenetic factors

The development of NAFLD is a complex process which includes genetic susceptibility (Brunt et al., 2015). Evidence of increased heritable risk of hepatic fat accumulation and mutations in metabolic processes have been shown in various genome-wide association studies (GWAS), candidate gene studies, and epigenetic studies (Brunt et al., 2015). Multiple loci have been identified that play a role in the regulation of metabolic processes including lipid and iron metabolism, inflammation insulin signaling, and fibrogenesis. Replicated evidence has shown 4 distinct loci, *PNPLA3*, *TM6SF2*, *INFL3*, and *GCKR*, associated with NAFLD. Patatin-like phospholipase domain-containing protein 3 (*PNPLA3*) is one such gene that has not only been linked to greater risk of progressive steatohepatitis and fibrosis but also of hepatocellular carcinoma (Liu et al., 2014). Genetic variation in proteins coded in *TM6SF2* (transmembrane 6 superfamily member 2) gene has been shown to increase blood lipid levels and has been associated with prevalence of both NAFLD and NASH (Pirola and Sookoian, 2015; Sookoian and Pirola, 2017). Variance in the *GCKR* locus (glucokinase regulatory gene) has been known to be involved in insulin sensitivity and has been linked to maturity-onset diabetes in young people (Dimas et al., 2014; Sookoian and Pirola, 2017). Polymorphisms in the interferon lambda 3 (*INFL3*) gene have been reported to be associated with increased hepatic inflammation and fibrosis in lean individuals with NAFLD (de Bruin et al., 2004).

Emerging evidence also links the development of NAFLD to regulation by microRNAs, which are short non-coding RNA species that regulate the degradation or translation of mRNA thereby regulating expression of myriad genes and pathways (Zarrinpar et al., 2016; Gjorgjieva et al., 2019; Lin et al., 2020; Sodum et al., 2021; Szabo, 2021). There is growing evidence for a role of microRNAs in the development of psychiatric disorders as well (Alural et al., 2017); however, data linking miRNA regulation of coincident NAFLD and mood disorders are limited. The species miR-34a has been implicated in the regulation of genes associated with hepatic lipid metabolism and secretion (Zarrinpar et al., 2016) and also in processes associated with synaptogenesis (Soto-Angona et al., 2020), and expression is altered in bipolar disorder (Alural et al., 2017). Additional research is needed to identify microRNA expression signatures that link metabolic liver disease with psychiatric disorders. Finally, microRNAs are being explored as potential diagnostic biomarkers and therapeutics for NAFLD/NASH (Di Mauro et al., 2021; Mohamed et al., 2021) and myriad psychiatric disorders as well (Alural et al., 2017; Roy et al., 2020; Islam et al., 2021; Srivastava et al., 2021; Tsermpini et al., 2022).

### Lean non-alcoholic fatty liver disease

Although most often comorbid with obesity, metabolic liver disease can occur in patients with normal body weight/BMI, termed lean NAFLD (LNAFLD). Although the pathophysiology of LNAFLD is not fully understood, it is an emerging medical problem that can be challenging to diagnose (Ye et al., 2020). Patients with LNAFLD tend to be younger in age, mostly male, and are less likely to present overt symptoms associated with the metabolic syndrome compared to NAFLD associated with obesity (Chen et al., 2020; Maier et al., 2021). The prevalence of LNAFLD is variable across populations, ranging from 12 to 23% (Kim et al., 2004; Chen et al., 2006; DiStefano and Gerhard, 2022). Histological comparison of obese and lean groups reveals similar grade of steatosis but the grade of necroinflammatory activity and stage of fibrosis were less advanced in lean than in obese patients (Leung et al., 2017).

The prevalence of NAFLD in patients with mental illness *in the absence of obesity* is not known, largely due to its diagnosis often being incidental. A recent report indicated that perceived stress was a significant, independent risk factor for NAFLD prevalence in apparently healthy women and men, including non-obese cohorts (Kang et al., 2020). Preclinical studies have reported that antipsychotic medications can induce NAFLD in the absence of weight gain (Rostama et al., 2020). Given the increased morbidity and mortality associated with NAFLD, regardless of BMI, additional studies are needed to examine the prevalence of metabolic liver disease in psychiatric patients who are non-obese.

### Metabolic liver disease and mental illness: Non-alcoholic fatty liver disease and the microbiome

The role of the gut microbiome in mental health and metabolic function has grown in appreciation in recent years. Microbes in the gastrointestinal tract aid in digestion, drug metabolism, and mucosal barrier integrity while rheostatically regulating local and systemic inflammation. Perturbations of the microbiome (i.e., dysbiosis) are both causes and results of disrupted homeostasis. NAFLD patients with and without psychiatric comorbidities have alterations in their gut microbiomes, often reciprocally linked to altered inflammatory responses and to metabolic syndrome (Harte et al., 2010; Solas et al., 2017). A direct role for gut dysbiosis in the pathogenesis of NAFLD was demonstrated using a preclinical murine model wherein fecal microbiota transplants (FMT) from donor mice with NAFLD were able to induce disease in germ-free recipient mice independent of obesity phenotype (Le Roy et al., 2013). Similarly, pre-, pro-, and syn-biotic supplementation has been shown to improve NAFLD status in rodent models and in patients (Cho et al., 2018).

The mechanisms by which gut dysbiosis leads to or exacerbates NAFLD are not clear, and are likely multi-faceted. Because the gut microbiome plays a central role in digestion and drug metabolism, changes in its composition can have profound effects on the gut metabolome. The gut-liver axis features blood flow from the small intestine and the colon to the liver, where exposure to potentially inflammatory metabolites such as phenols, ethanol, acetaldehyde, ammonia, and secondary bile acids from the gut is very high (de Bruin et al., 2004; Zhu et al., 2013; Safari and Gérard, 2019; Chen et al., 2020). Dysbiotic gut microbiomes have also been shown to alter lipid metabolism in the liver, potentiating additional adiposity and lipid peroxidation (Solas et al., 2017). Finally, dysbiosis can lead to disruption of the endothelial barrier of the gut, a phenomenon known as leaky gut syndrome. Leaky gut syndrome allows for the release of pro-inflammatory microbial products such as lipopolysaccharide (LPS) into the bloodstream, wherein they immediately cross the gut-liver access and potentiate hepatic inflammation (Kessoku et al., 2021).

The dysbiotic signatures associated with NAFLD are somewhat variable across studies; however, general patterns of decreased microbial diversity and disrupted *Bacillota* to *Bacteroidota* (previously known as *Firmicutes* and *Bacteroidetes*, respectively) ratio relative to healthy control patients is common. An additional trend evident in NAFLD/NASH patients is an increase in *Pseudomonadota* (formerly *Proteobacteria*) species, particularly members of the *Enterobacteriaceae* (Reviewed in references (Solas et al., 2017; Cho et al., 2018). Taken together, the overall pattern of dysbiosis appears to be a decrease in species richness and a concurrent increase in the abundance of Gram negative bacteria relative to Gram positives. These findings are consistent with a contribution of leaky gut syndrome to the pathogenesis of NAFLD/NASH, because an increased abundance of Gram negative organisms would substantially increase the release of LPS into the blood. NAFLD and NASH patients are reported to experience endotoxemia i.e., the detection of LPS in the blood; (Kessoku et al., 2021), which is also consistent with elevated *Bacteroidota* and *Pseudomonota* presence. Additionally, probiotic supplementations that are most associated with amelioration of NAFLD in patients and preclinical models all include members of the phylum *Bacillota*, likely representing partial restoration of a healthy gut microbiome (Cho et al., 2018; Safari and Gérard, 2019).

Altered inflammatory profiles and gut dysbiosis have both been reported in psychiatric patients and patients taking AA medications, as well as animal models of cognitive dysfunction and drug exposure (Schuld et al., 2004; Drzyzga et al., 2006; Pyndt Jørgensen et al., 2015; Flowers et al., 2017; May et al., 2019, 2020; Zheng et al., 2019; Zeng et al., 2021). As seen with NAFLD, psychiatric patients exhibit a general trend toward decreased bacterial diversity, lower abundance of *Bacillota* species, and increased prevalence of *Pseudomonatota* species. Different dysbiotic gut signatures were also shown to correlate with resistance to antipsychotic treatment in schizophrenia patients, suggesting that the gut microbiome can impact treatment efficacy (Schwarz et al., 2018). Interpretation of changes in gut microbiomes associated with antipsychotic medication use in patients are somewhat confounded by changes seen in unmedicated psychiatric patients. However, a rodent model of olanzapine treatment showed an increase in the *Bacillota* to *Bacteroidota* ratio that is the inverse to that seen in both psychiatric patients and NAFLD patients. However, this ratio is still dysbiotic, as treated animals experienced metabolic disruption and weight gain (Davey et al., 2012; Skonieczna-Żydecka et al., 2019). Both the weight gain and metabolic syndrome were reversed with either probiotic (Dhaliwal et al., 2019; Huang D. et al., 2021) or antibiotic (Davey et al., 2013) therapy, mechanistically linking them to gut microbiome composition. These findings suggest that the disruption of the gut microbiome may contribute to the pathogenesis of both NAFLD and psychosis through complex interacts of dysregulated inflammation, leaky gut syndrome, and metabolic disruption. These findings also suggest that: (1) the dysbiosis induced by AA medications in otherwise healthy animals may reflect an attempt to restore gut homeostasis from the dysbiosis characteristic of schizophrenia and bipolar disorder; and (2) NAFLD associated with antipsychotic medication use likely stems from dysregulated inflammation and metabolic disruption, but not leaky gut syndrome.

### Nicotine consumption

Smoking leads to preventable morbidities such as heart disease, diabetes and cancer (Samet, 2013; Maddatu et al., 2017; Münzel et al., 2020). Smoking is also highly prevalent among those with mental health disorders including schizophrenia (three times higher than population norms) and bipolar disorder (two times higher) (de Leon and Diaz, 2012; Jackson et al., 2015; Dickerson et al., 2018). Nicotine consumed from smoking cigarettes or electronic cigarettes has been linked to insulin resistance, type 2 diabetes and increased cardiovascular risk (Willi et al., 2007; Bajaj, 2012; Maddatu et al., 2017; El-Mahdy et al., 2021). Nicotine binds to nicotinic acetylcholine α1 receptors, inhibiting hypothalamic AMP-activated protein kinase (AMPK) activity leading to reduced food intake and increased whole body thermogenesis, coincident with increasing lipolysis and free fatty acid delivery to the liver and skeletal muscle. As a result, hepatic VLDL secretion intramyocellular lipid saturation is increased. All factors combined contribute to increased insulin resistance and metabolic disease risk (Bajaj, 2012). Additionally, smoking accelerates hepatic fibrosis and has been linked to the development of hepatocellular carcinoma (Koh et al., 2011). Nicotine directly activates stellate cells, leading to fibrosis while indirectly activating the pro-inflammatory cytokines IL-6, IL8 and TNF alpha and leading to hepatic injury. A meta-analysis of epidemiologic studies consisting of 38 cohort studies and 58 case studies demonstrated that cigarette smoking increased the rate of hepatocellular carcinoma after adjusting for hepatitis B, hepatitis C, and alcohol consumption. Compared with people who have never smoked, the adjusted mortality risk ratio for current smokers was 1.51 [95% confidence interval (CI) 1.37–1.67] while former smokers were 1.12 (95% CI 0.78–1.60); (Lee et al., 2009).

As smoking has a higher prevalence among patients with psychiatric disorders and leads to insulin resistance, it is likely that nicotine consumption contributes to the increased incidence of NAFLD in this patient population. In support of this hypothesis, Hamabe et al. (2011) conducted a retrospective study consisting of 2,029 participants who underwent a complete medical health checkup in the span of 10 years including screening for NAFLD. After adjusting for Hepatitis B, Hepatitis C, and alcohol consumption, 17.1% of participants were found to have developed NAFLD in a 10-year period with cigarette smoking as a risk factor independent of metabolic syndrome risk. Similarly, NAFLD incidence was increased in a cohort of Japanese patients who smoked, but did not consume alcohol (Okamoto et al., 2018) and cotinine-verified current smoking and self-reported current smoking status were shown to be independent risk factors for NAFLD (Kim N. H. et al., 2018).

**FIGURE 6:**
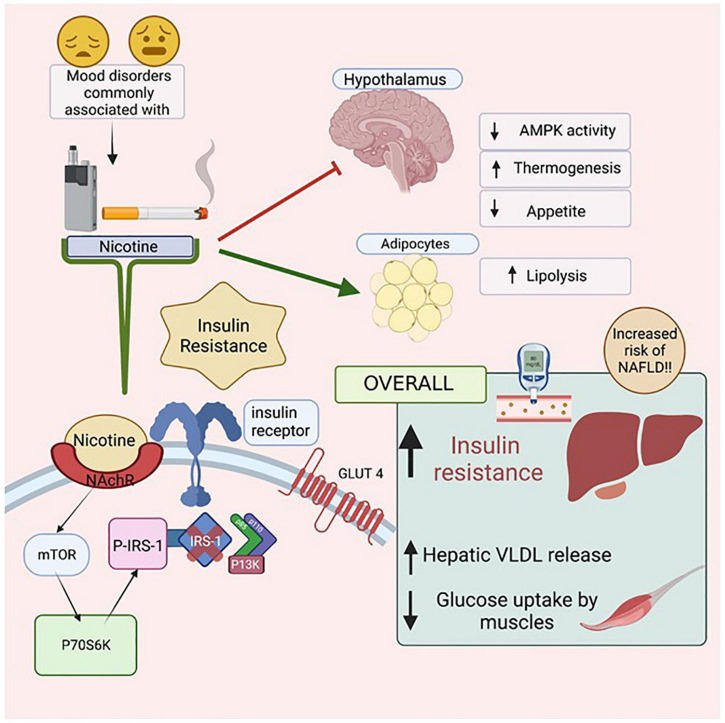
The Effects of Nicotine on insulin resistance and NAFLD. Mental health illnesses, including mood disorders, have high co-incidence rates with smoking. Nicotine, found in both cigarettes and electronic vapes, has been linked to increasing insulin resistance. When Nicotine binds to its receptor, it activates mTOR which then activates P70S6K which activates P-IRS-1, preventing Insulin Receptor Substrate (IRS-1) from binding to the cytoplasmic domain of the insulin receptors. As a result, intracellular pathway PI3K will not be activated resulting in eventual insulin resistance. Due to such reactions in the Hypothalamus, there is a decrease in AMPK activity, increased thermogenesis and reduced appetite. In adipocytes, there is an increase in lipolysis, leading to increased levels of free fatty acids in the body. Figure created with BioRender.com.

Nicotine use is prevalent in patients with mental illness, leads to insulin resistance and emerging evidence links nicotine use to the development and progression of NAFLD (Figure 6). Further understanding of the complex relationship between nicotine, mental health, insulin resistance, and NAFLD will help inform preventative screening procedures and treatment for high risk patient populations.

### Social determinants of health

NAFLD/NASH prevalence is epidemiologically linked to multiple social determinants of health, including poverty, food or housing insecurity, and poor access to healthcare (Talens et al., 2021). Patients experiencing poor determinants of health also have a higher prevalence of NAFLD-associated traits including insulin resistance, obesity, and metabolic syndrome (Cho et al., 2021). Lack of access to safe and nutritious food was directly determined to be an independent risk factor for developing NAFLD (Golovaty et al., 2020), implicating lack of food security in the constellation of risk (Talens et al., 2021).

Higher incidence of psychiatric conditions also correlates with lower socioeconomic status (Hudson, 2005; Roy-Byrne et al., 2009). A significantly larger proportion of AA prescriptions were to those who had Medicaid as a primary source of payment (Leckman-Westin et al., 2018). Compared with children eligible for income-based Medicaid, children receiving supplemental security income and those in foster care are twice as likely to receive higher than recommended doses of AA medications (Leckman-Westin et al., 2018). Because mental illness is an independent risk factor of NAFLD in adults and children, and both conditions correlate inversely with socioeconomic status, social determinants of health likely contribute to the burden of mental illness and metabolic disease in vulnerable populations. Public health policy as well as development of improved diagnostics and targeted NAFLD therapeutics are needed to address the complexity of metabolic liver disease in vulnerable populations.

### Linkage between climate change, non-alcoholic fatty liver disease, and mental health

The intersection between climate change and NAFLD is underappreciated but becoming increasingly relevant. Agricultural infrastructure set up to provide food and other resources has been collapsing as scarcity of available land, drought, wildfires, and the frequency of major storms directly influence farming (Donnelly et al., 2022). This in turn contributes to increasing rates of obesity as more people increasingly rely on processed foods (Donnelly et al., 2022). Obesity has been linked to development of insulin resistance, and both contribute to the development of NAFLD.

Changes in environmental conditions, such as those associated with climate change, have been shown to alter or entrain circadian rhythms in diverse species (Prokkola and Nikinmaa, 2018), and it is clear that the circadian clock is heavily involved in regulating metabolic homeostasis, mood, behavioral processes, and medication effects. When there are changes or disruptions to the circadian rhythm, there are consequential shifts in varied but interconnected physiological systems. The impact of climate change-associated alterations in light: dark exposures on the incidence of both NAFLD and mental health disorders is an area where prospective evaluations are urgently needed.

In addition to dwindling resources resulting in food insecurity, the ecological impacts brought about by climate change can have a negative effect on mental health and spirituality. This has been noted particularly in indigenous communities, where an increased prevalence of mental health disorders coincident with climate change has been attributed to the concept of ecological grief (Middleton et al., 2020; Lebel et al., 2022). Consistent with the correlative pattern and potential mechanistic overlap between the two conditions, NAFLD prevalence in an indigenous population was far higher than the global rate (44 vs. 32%, respectively), and indigenous communities were identified as one of the groups with the steepest increases in rates of NAFLD-related mortality over the past 10 years (Cheah et al., 2013; Paik et al., 2019; Riazi et al., 2022).

NAFLD, mental health, and climate change have a multi-factorial intersection, and understanding of their interactions and impacts on human health are nascent. Future studies to ascertain how geographical location impacts the incidence of NAFLD, as well as how our rapidly changing global climate impacts the prevalence of metabolic disease and mental illness are highly warranted.

## Summary and future directions

In summary, NAFLD/NASH incidence is growing world-wide and is highly comorbid with mental illness (Box 1). As patients with mental illness are at increased risk for development of NAFLD, clinical guidelines are needed which specifically inform patient monitoring and psychiatric medication prescribing practice as it relates to metabolic liver disease. Furthermore, despite evidence that psychiatric medications such as AA contribute to NAFLD/NASH incidence, currently clinical data are lacking that define relative NAFLD/NASH risk for specific psychiatric medications (and corresponding mechanistic toxicology mechanisms). This is an important area for future research efforts, in order to better inform clinical prescribing practice and patient monitoring.

**Box 1. Key Points**

• NAFLD/NASH is highly comorbid with psychiatric disorders and can have myriad intersecting genetic and environmental causes
• Smoking, sedentary lifestyle, high fat/obesogenic diets, food insecurity and other socioeconomic factors contribute to incidence of NAFLD in patients with mental illness
• Insulin resistance, with or without obesity, increases the incidence of NAFLD/NASH
• Systemic inflammation, characteristic of chronic diseases such as obesity and diabetes, contributes to both mood disorders and NAFLD/NASH
• Psychiatric medications including SSRI antidepressants and atypical antipsychotics are associated with increased risk for NAFLD/NASH
• Psychiatric medications increase NAFLD/NASH through diverse pharmacological mechanisms, some of which are distinct from medication associated insulin resistance

As diagnosis is hampered by lack of robust, accessible clinical biomarkers and as there are currently no FDA-approved therapies for the treatment of NAFLD, additional research is needed to provide clinicians with robust diagnostic and therapeutic tools to enable patient care. A promising addition to the diagnostic arsenal is C-reactive protein (CRP) intervention. CRP is an easily detected marker of inflammation; however, its use for medical decision-making has been historically controversial (Santos et al., 2012). The benefits of rapid or bedside CRP measurement are most clear for acute inflammatory conditions such as bacterial infection. CRP levels are strong predictors of bacterial infection in pediatric patients and patients with fever of unknown origin, and clinical decision-making based on bedside CRP measures significantly lowered length of hospital stays (Olaciregui et al., 2009; Nijman et al., 2014, 2015). High-sensitivity CRP measurements (hsCRP), which have a higher diagnostic sensitivity for inflammation than standard CRP tests, have significant potential to act as a pre-biopsy proxy for NAFLD. Significant correlations between NAFLD and hsCRP levels independent of obesity and metabolic syndrome have been reported (Ndumele et al., 2011; Kumar et al., 2020), and high or high/normal hsCRP levels were predictive of subsequent NAFLD diagnosis (Lee et al., 2017). In contrast, earlier studies reported that hsCRP does not accurately discriminate between NAFLD and NASH; however, there is an important role for this measure in identifying patients at risk for NAFLD or NASH as opposed to healthy controls (Hui et al., 2004). It is notable that none of the currently accepted diagnostic matrices (i.e., NLFS, HIS, FLI), which boast diagnostic sensitivity of 70–80%, incorporate CRP or hsCRP. Inclusion of hsCRP, which is positively correlated with NAFLD and NASH diagnoses, is likely to significantly strengthen to non-invasive diagnostic power of any or all of these matrices for patients with NAFLD.

Multiple drug classes currently in Phase III clinical development for NAFLD/NASH target the diverse pathophysiology associated with metabolic liver disease including fibroblast growth factor analogs, PPAR transcription factors, bile acid receptors, SGLT2 inhibitors (Ala, 2021), GLP-1 receptors and thyroid hormone receptors (Sinakos et al., 2022). Among currently FDA approved medications (for obesity and/or diabetes treatment), GLP-1 receptor agonists appear especially promising for the treatment of NAFLD (Mahapatra et al., 2022; Patel Chavez et al., 2022). Given the significant impact of psychiatric medications on whole-body metabolism and NAFLD, alternative therapies/co-therapies are important therapeutic approaches for psychiatry. Recent FDA approval of a combination therapeutic, olanzapine/sandorphin (LYBALVI™) focused on minimizing olanzapine associated weight gain, illustrates new approaches to minimize metabolic side effects of psychiatric medications that are central to psychiatric disease management (Monahan et al., 2022).

## Author contributions

AG contributed to writing the manuscript and figure creation. RI and KT contributed to writing the manuscript. MM contributed to writing and editing the manuscript and figure creation. KH contributed to manuscript concept development, writing, figure creation, and editing. All authors contributed to the article and approved the submitted version.
